# 4-Amino­pyridinium 2-hy­droxy­benzoate

**DOI:** 10.1107/S1600536810027042

**Published:** 2010-07-14

**Authors:** Hoong-Kun Fun, Madhukar Hemamalini, Venkatachalam Rajakannan

**Affiliations:** a X-ray Crystallography Unit, School of Physics, Universiti Sains Malaysia, 11800 USM, Penang, Malaysia; b Biomedical Structural Biology, School of Biological Sciences, Nanyang Technological University, Singapore 138673

## Abstract

In the salicylate anion of the title salt, C_5_H_7_N_2_^+^·C_7_H_5_O_3_^−^, an intra­molecular O—H⋯O hydrogen bond generating an *S*(6) ring motif is observed. In the crystal structure, the cations and anions are linked into a two-dimensional network parallel to the *ab* plane by N—H⋯O and C—H⋯O hydrogen bonds. The network contains *R*_2_^2^(7) and *R*_1_^2^(4) ring motifs. Weak π–π inter­actions between the benzene and pyridinium rings [centroid–centroid distance = 3.688 (1) Å] are also observed.

## Related literature

For the biological activity of 4-amino­pyridine, see: Schwid *et al.* (1997 ▶). For the crystal structure of 4-amino­pyridine, see: Chao & Schempp (1977 ▶); Anderson *et al.* (2005 ▶). For related structures, see: Bhattacharya *et al.* (1994 ▶); Karle *et al.* (2003 ▶); Gellert & Hsu (1988 ▶); Hemamalini & Fun (2010 ▶). For hydrogen-bond motifs, see: Bernstein *et al.* (1995 ▶). For bond-length data, see: Allen *et al.* (1987 ▶). For the stability of the temperature controller used in the data collection, see: Cosier & Glazer (1986 ▶).
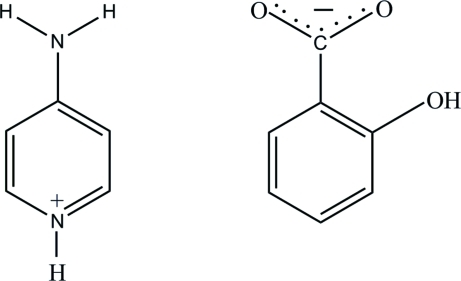


## Experimental

### 

#### Crystal data

C_5_H_7_N_2_^+^·C_7_H_5_O_3_^−^
*M* *_r_* = 232.24
Orthorhombic, 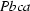

*a* = 12.5801 (2) Å
*b* = 11.4157 (2) Å
*c* = 15.7560 (3) Å
*V* = 2262.73 (7) Å^3^
*Z* = 8
Mo *K*α radiation
μ = 0.10 mm^−1^
*T* = 100 K
0.29 × 0.17 × 0.08 mm

#### Data collection

Bruker SMART APEXII CCD area-detector diffractometer
Absorption correction: multi-scan (*SADABS*; Bruker, 2009 ▶) *T*_min_ = 0.971, *T*_max_ = 0.992
15672 measured reflections
3010 independent reflections
2303 reflections with *I* > 2σ(*I*)
*R*_int_ = 0.057

#### Refinement

*R*[*F* ^2^ > 2σ(*F* ^2^)] = 0.059
*wR*(*F* ^2^) = 0.118
*S* = 1.09
3010 reflections
170 parameters
H atoms treated by a mixture of independent and constrained refinement
Δρ_max_ = 0.37 e Å^−3^
Δρ_min_ = −0.26 e Å^−3^

### 

Data collection: *APEX2* (Bruker, 2009 ▶); cell refinement: *SAINT* (Bruker, 2009 ▶); data reduction: *SAINT*; program(s) used to solve structure: *SHELXTL* (Sheldrick, 2008 ▶); program(s) used to refine structure: *SHELXTL*; molecular graphics: *SHELXTL*; software used to prepare material for publication: *SHELXTL* and *PLATON* (Spek, 2009 ▶).

## Supplementary Material

Crystal structure: contains datablocks global, I. DOI: 10.1107/S1600536810027042/ci5130sup1.cif

Structure factors: contains datablocks I. DOI: 10.1107/S1600536810027042/ci5130Isup2.hkl

Additional supplementary materials:  crystallographic information; 3D view; checkCIF report

## Figures and Tables

**Table 1:** Hydrogen-bond geometry (Å, °)

*D*—H⋯*A*	*D*—H	H⋯*A*	*D*⋯*A*	*D*—H⋯*A*
N1—H1*N*1⋯O2^i^	0.96 (2)	2.48 (2)	3.1394 (19)	126 (2)
N1—H1*N*1⋯O3^i^	0.96 (2)	1.78 (2)	2.7296 (19)	172 (2)
N2—H1*N*2⋯O2	0.89 (2)	1.90 (2)	2.789 (2)	176 (2)
O1—H1*O*1⋯O3	0.97 (3)	1.61 (2)	2.5316 (18)	157 (2)
C11—H11*A*⋯O3^ii^	0.93	2.55	3.360 (2)	146
C12—H12*A*⋯O2^i^	0.93	2.56	3.164 (2)	123

Symmetry codes: (i) 
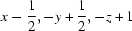
; (ii) 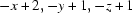.

**Fig. 1.**
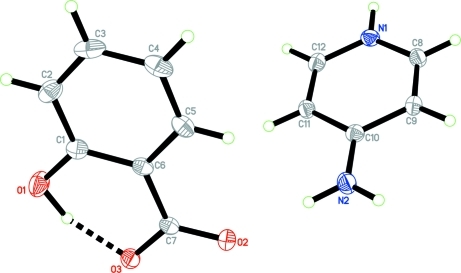
The asymmetric unit of the title compound. Displacement ellipsoids are drawn at the 50% probability level. Dashed line indicates the intramolecular hydrogen bond.

**Fig. 2.**
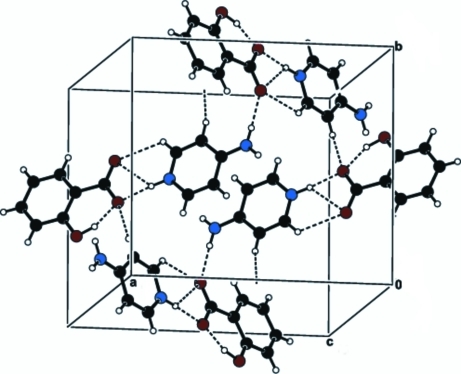
The crystal packing of the title compound, showing a hydrogen-bonded (dashed lines) 2D network.

## supplementary crystallographic information

### Comment

Aminopyridines are key intermediates for the synthesis of important pharmaceuticals and agrochemicals. Particularly, 4-aminopyridine (fampridine) is used in the treatment of neurological ailments, such as multiple sclerosis (MS), with tests showing that fampridine improves motor function in MS patients (Schwid *et al.*, 1997). The crystal structure of 4-amino pyridine was first reported by Chao and Schempp (1977) and a redetermination was reported by Anderson *et al.* (2005). Salicylic acid (SA) is a common component in liquid scintillation systems. Salts of salicylic acid are good candidates for dry solid scintillators. Knowledge of these structural data is important to the development of a fundamental understanding of its scintillating properties, and more generally a predictive capability for tailoring materials to achieve desired scintillation properties. The present study has been carried out in order to study the hydrogen bonding patterns present in the crystal structure of 4-aminopyridinium salicylate, (I).

The asymmetric unit of (I) (Fig. 1) contains one 4-aminopyridinium cation and one salicylate anion, indicating that proton transfer occurred during the co-crystallisation experiment. Protonation leads to the widening of C8—N1—C12 angle in the pyridine ring to 120.26 (16)°, compared to 115.25 (13)° in neutal 4-aminopyridine (Anderson *et al.*, 2005). This type of protonation has been observed in various 4-aminopyridine acid complexes (Bhattacharya *et al.*, 1994; Karle *et al.*, 2003). The bond lengths (Allen *et al.*, 1987) and angles are within normal ranges.

In the crystal packing (Fig. 2), the protonated N atom and the hydrogen atom attached to atom C12 are hydrogen-bonded to the carboxylate oxygen atoms (O2 and O3) via N1—H1N1···O3 and C12—H12A···O2 hydrogen bonds, leading to the formation of an *R*^2^_2_(7) ring motif (Bernstein *et al.*, 1995). The carboxylate O atoms of the salicylate anion act as acceptors of bifurcated N1—H1N1···O2 and N1—H1N1···O3 hydrogen bonds with the protonated aromatic ring N atom of the 4-aminopyridinium cation, forming a ring with the graph-set notation *R*^2^_1_(4).  Furthermore, these two motifs are connected via N2—H1N2···O2 and C11—H11A···O3 (Table 1) hydrogen bonds, forming a two-dimensional network parallel to the *ab*-plane. There is an intramolecular O1—H1O1···O3 hydrogen bond in the salicylate anion, which generates an *S*(6) ring motif. This motif is also observed in the crystal structures of 2-aminopyridinium salicylate (Gellert & Hsu, 1988) and 2-amino-5-chloropyridinium salicylate (Hemamalini & Fun, 2010). The crystal structure is further stabilized by π–π interactions between the benzene ring at (x, y, z) and pyridinium ring at (3/2-x, 1/2+y, z) with a centroid-to-centroid distance of 3.688 (1) Å.

### Experimental

A hot methanol solution (20 ml) of 4-aminopyridine (0.04705 g, Aldrich) and salicylic acid (0.0691 g, Merck) was warmed for 30 min over a water bath. The solution was cooled slowly and kept at room temperature. After a few days, colourless crystals were obtained.

### Refinement

Atoms H1N1, H1N2, H2N2 and H1O1 were located from a difference Fourier map and were refined freely [N–H= 0.86 (2)–0.96 (2) Å and O–H = 0.97 (3) Å]. The remaining H atoms were positioned geometrically [C–H = 0.93 Å] and were refined using a riding model, with *U*_iso_(H) = 1.2 *U*_eq_(C).

### Crystal data

**Table:**

C_5_H_7_N_2_^+^·C_7_H_5_O_3_^−^	*F*(000) = 976
*M**_r_* = 232.24	*D*_x_ = 1.363 Mg m^−^^3^
Orthorhombic, *P**b**c**a*	Mo *K*α radiation, λ = 0.71073 Å
Hall symbol: -P 2ac 2ab	Cell parameters from 2403 reflections
*a* = 12.5801 (2) Å	θ = 2.6–28.5°
*b* = 11.4157 (2) Å	µ = 0.10 mm^−^^1^
*c* = 15.7560 (3) Å	*T* = 100 K
*V* = 2262.73 (7) Å^3^	Plate, colourless
*Z* = 8	0.29 × 0.17 × 0.08 mm

### Data collection

**Table:**

Bruker SMART APEXII CCD area-detector diffractometer	3010 independent reflections
Radiation source: fine-focus sealed tube	2303 reflections with *I* > 2σ(*I*)
graphite	*R*_int_ = 0.057
φ and ω scans	θ_max_ = 29.0°, θ_min_ = 2.6°
Absorption correction: multi-scan (*SADABS*; Bruker, 2009)	*h* = −17→11
*T*_min_ = 0.971, *T*_max_ = 0.992	*k* = −15→15
15672 measured reflections	*l* = −21→16

### Refinement

**Table:**

Refinement on *F*^2^	Primary atom site location: structure-invariant direct methods
Least-squares matrix: full	Secondary atom site location: difference Fourier map
*R*[*F*^2^ > 2σ(*F*^2^)] = 0.059	Hydrogen site location: inferred from neighbouring sites
*wR*(*F*^2^) = 0.118	H atoms treated by a mixture of independent and constrained refinement
*S* = 1.09	*w* = 1/[σ^2^(*F*_o_^2^) + (0.0323*P*)^2^ + 1.7134*P*] where *P* = (*F*_o_^2^ + 2*F*_c_^2^)/3
3010 reflections	(Δ/σ)_max_ = 0.001
170 parameters	Δρ_max_ = 0.37 e Å^−^^3^
0 restraints	Δρ_min_ = −0.26 e Å^−^^3^

### Special details

**Table:**

Experimental. The crystal was placed in the cold stream of an Oxford Cryosystems Cobra open-flow nitrogen cryostat (Cosier & Glazer, 1986) operating at 100.0 (1) K.
Geometry. All s.u.'s (except the s.u. in the dihedral angle between two l.s. planes) are estimated using the full covariance matrix. The cell s.u.'s are taken into account individually in the estimation of s.u.'s in distances, angles and torsion angles; correlations between s.u.'s in cell parameters are only used when they are defined by crystal symmetry. An approximate (isotropic) treatment of cell s.u.'s is used for estimating s.u.'s involving l.s. planes.
Refinement. Refinement of F^2^ against ALL reflections. The weighted R-factor wR and goodness of fit S are based on F^2^, conventional R-factors R are based on F, with F set to zero for negative F^2^. The threshold expression of F^2^ > 2σ(F^2^) is used only for calculating R-factors(gt) etc. and is not relevant to the choice of reflections for refinement. R-factors based on F^2^ are statistically about twice as large as those based on F, and R- factors based on ALL data will be even larger.

### Fractional atomic coordinates and isotropic or equivalent isotropic displacement parameters (Å^2^)

**Table:**

	*x*	*y*	*z*	*U*_iso_*/*U*_eq_	
O1	0.93694 (11)	0.70642 (11)	0.39307 (9)	0.0267 (3)	
O2	1.08510 (10)	0.38258 (10)	0.38336 (8)	0.0216 (3)	
O3	1.08570 (10)	0.56634 (10)	0.43059 (8)	0.0204 (3)	
C1	0.89745 (14)	0.62078 (15)	0.34345 (11)	0.0184 (4)	
C2	0.80559 (14)	0.64452 (17)	0.29653 (12)	0.0227 (4)	
H2A	0.7739	0.7180	0.2999	0.027*	
C3	0.76181 (15)	0.55938 (18)	0.24519 (12)	0.0255 (4)	
H3A	0.7003	0.5757	0.2146	0.031*	
C4	0.80871 (15)	0.44924 (17)	0.23863 (12)	0.0242 (4)	
H4A	0.7792	0.3923	0.2036	0.029*	
C5	0.89968 (14)	0.42548 (16)	0.28476 (11)	0.0203 (4)	
H5A	0.9312	0.3520	0.2802	0.024*	
C6	0.94544 (13)	0.50956 (15)	0.33810 (11)	0.0163 (3)	
C7	1.04482 (14)	0.48238 (15)	0.38720 (11)	0.0164 (3)	
N1	0.76523 (12)	0.02312 (13)	0.49686 (9)	0.0182 (3)	
N2	1.02794 (13)	0.14710 (14)	0.37198 (11)	0.0217 (3)	
C8	0.82546 (14)	−0.05055 (15)	0.45039 (11)	0.0186 (4)	
H8A	0.8061	−0.1290	0.4468	0.022*	
C9	0.91367 (14)	−0.01310 (15)	0.40865 (11)	0.0178 (4)	
H9A	0.9543	−0.0655	0.3772	0.021*	
C10	0.94313 (13)	0.10635 (14)	0.41341 (11)	0.0162 (3)	
C11	0.87938 (14)	0.18104 (15)	0.46358 (11)	0.0170 (4)	
H11A	0.8970	0.2598	0.4692	0.020*	
C12	0.79224 (14)	0.13756 (15)	0.50369 (11)	0.0186 (4)	
H12A	0.7504	0.1873	0.5364	0.022*	
H1N1	0.7029 (19)	−0.003 (2)	0.5260 (15)	0.039 (7)*	
H2N2	1.0665 (18)	0.102 (2)	0.3412 (15)	0.032 (6)*	
H1N2	1.0479 (19)	0.222 (2)	0.3735 (15)	0.042 (7)*	
H1O1	1.000 (2)	0.669 (2)	0.4166 (18)	0.061 (9)*	

### Atomic displacement parameters (Å^2^)

**Table:**

	*U*^11^	*U*^22^	*U*^33^	*U*^12^	*U*^13^	*U*^23^
O1	0.0301 (8)	0.0199 (7)	0.0302 (8)	0.0066 (6)	−0.0086 (6)	−0.0064 (5)
O2	0.0199 (6)	0.0150 (6)	0.0297 (7)	0.0019 (5)	−0.0014 (6)	−0.0015 (5)
O3	0.0196 (6)	0.0173 (6)	0.0242 (7)	−0.0002 (5)	−0.0051 (5)	−0.0032 (5)
C1	0.0177 (8)	0.0214 (9)	0.0162 (9)	−0.0010 (7)	0.0016 (7)	−0.0002 (7)
C2	0.0191 (9)	0.0280 (9)	0.0210 (10)	0.0050 (8)	0.0032 (7)	0.0042 (7)
C3	0.0151 (9)	0.0420 (11)	0.0195 (9)	−0.0039 (8)	−0.0022 (7)	0.0081 (8)
C4	0.0243 (10)	0.0305 (10)	0.0179 (9)	−0.0106 (8)	−0.0027 (8)	0.0005 (8)
C5	0.0224 (9)	0.0200 (8)	0.0184 (9)	−0.0060 (7)	0.0012 (7)	0.0008 (7)
C6	0.0151 (8)	0.0193 (8)	0.0146 (8)	−0.0035 (7)	0.0016 (6)	0.0002 (6)
C7	0.0154 (8)	0.0176 (8)	0.0162 (8)	−0.0026 (7)	0.0014 (7)	0.0006 (6)
N1	0.0153 (7)	0.0189 (7)	0.0205 (8)	−0.0016 (6)	0.0007 (6)	0.0020 (6)
N2	0.0219 (8)	0.0166 (8)	0.0265 (9)	−0.0017 (7)	0.0073 (7)	−0.0021 (6)
C8	0.0201 (9)	0.0148 (8)	0.0209 (9)	−0.0010 (7)	−0.0027 (7)	0.0003 (7)
C9	0.0195 (8)	0.0149 (8)	0.0190 (9)	0.0021 (7)	0.0005 (7)	−0.0014 (6)
C10	0.0158 (8)	0.0174 (8)	0.0155 (8)	0.0003 (6)	−0.0020 (7)	0.0014 (6)
C11	0.0191 (9)	0.0147 (8)	0.0171 (9)	0.0006 (7)	−0.0025 (7)	−0.0009 (6)
C12	0.0200 (9)	0.0183 (8)	0.0175 (9)	0.0036 (7)	−0.0009 (7)	−0.0016 (7)

### Geometric parameters (Å, °)

**Table:**

O1—C1	1.347 (2)	N1—C8	1.348 (2)
O1—H1O1	0.97 (3)	N1—C12	1.354 (2)
O2—C7	1.248 (2)	N1—H1N1	0.96 (2)
O3—C7	1.285 (2)	N2—C10	1.335 (2)
C1—C2	1.398 (3)	N2—H2N2	0.86 (2)
C1—C6	1.408 (2)	N2—H1N2	0.89 (3)
C2—C3	1.379 (3)	C8—C9	1.359 (2)
C2—H2A	0.93	C8—H8A	0.93
C3—C4	1.393 (3)	C9—C10	1.415 (2)
C3—H3A	0.93	C9—H9A	0.93
C4—C5	1.382 (3)	C10—C11	1.412 (2)
C4—H4A	0.93	C11—C12	1.359 (2)
C5—C6	1.400 (2)	C11—H11A	0.93
C5—H5A	0.93	C12—H12A	0.93
C6—C7	1.503 (2)		
			
C1—O1—H1O1	101.7 (16)	C8—N1—C12	120.26 (16)
O1—C1—C2	118.11 (16)	C8—N1—H1N1	122.0 (14)
O1—C1—C6	122.07 (16)	C12—N1—H1N1	117.7 (14)
C2—C1—C6	119.82 (16)	C10—N2—H2N2	121.1 (15)
C3—C2—C1	120.23 (17)	C10—N2—H1N2	123.1 (16)
C3—C2—H2A	119.9	H2N2—N2—H1N2	116 (2)
C1—C2—H2A	119.9	N1—C8—C9	121.71 (16)
C2—C3—C4	120.70 (18)	N1—C8—H8A	119.1
C2—C3—H3A	119.7	C9—C8—H8A	119.1
C4—C3—H3A	119.7	C8—C9—C10	119.43 (16)
C5—C4—C3	119.26 (17)	C8—C9—H9A	120.3
C5—C4—H4A	120.4	C10—C9—H9A	120.3
C3—C4—H4A	120.4	N2—C10—C11	121.15 (16)
C4—C5—C6	121.44 (17)	N2—C10—C9	121.29 (16)
C4—C5—H5A	119.3	C11—C10—C9	117.56 (16)
C6—C5—H5A	119.3	C12—C11—C10	119.85 (16)
C5—C6—C1	118.54 (16)	C12—C11—H11A	120.1
C5—C6—C7	120.65 (16)	C10—C11—H11A	120.1
C1—C6—C7	120.80 (15)	N1—C12—C11	121.17 (16)
O2—C7—O3	122.97 (16)	N1—C12—H12A	119.4
O2—C7—C6	120.08 (15)	C11—C12—H12A	119.4
O3—C7—C6	116.94 (15)		
			
O1—C1—C2—C3	−179.61 (17)	C1—C6—C7—O2	178.36 (16)
C6—C1—C2—C3	0.1 (3)	C5—C6—C7—O3	175.74 (16)
C1—C2—C3—C4	−0.6 (3)	C1—C6—C7—O3	−3.0 (2)
C2—C3—C4—C5	0.5 (3)	C12—N1—C8—C9	−0.6 (3)
C3—C4—C5—C6	0.2 (3)	N1—C8—C9—C10	−0.4 (3)
C4—C5—C6—C1	−0.7 (3)	C8—C9—C10—N2	−178.64 (17)
C4—C5—C6—C7	−179.52 (16)	C8—C9—C10—C11	1.3 (2)
O1—C1—C6—C5	−179.75 (16)	N2—C10—C11—C12	178.68 (17)
C2—C1—C6—C5	0.6 (2)	C9—C10—C11—C12	−1.2 (2)
O1—C1—C6—C7	−0.9 (3)	C8—N1—C12—C11	0.6 (3)
C2—C1—C6—C7	179.35 (16)	C10—C11—C12—N1	0.3 (3)
C5—C6—C7—O2	−2.9 (2)		

### Hydrogen-bond geometry (Å, °)

**Table:**

*D*—H···*A*	*D*—H	H···*A*	*D*···*A*	*D*—H···*A*
N1—H1N1···O2^i^	0.96 (2)	2.48 (2)	3.1394 (19)	126 (2)
N1—H1N1···O3^i^	0.96 (2)	1.78 (2)	2.7296 (19)	172 (2)
N2—H1N2···O2	0.89 (2)	1.90 (2)	2.789 (2)	176 (2)
O1—H1O1···O3	0.97 (3)	1.61 (2)	2.5316 (18)	157 (2)
C11—H11A···O3^ii^	0.93	2.55	3.360 (2)	146
C12—H12A···O2^i^	0.93	2.56	3.164 (2)	123

Symmetry codes: (i) *x*−1/2, −*y*+1/2, −*z*+1; (ii) −*x*+2, −*y*+1, −*z*+1.
